# Investigating the psychometric properties of the Patient Health Questionnaire for Adolescents (PHQ-A) and Center for Epidemiologic Studies - Depression Scale for Children (CES-DC) among young adolescents in South Africa

**DOI:** 10.1371/journal.pone.0334658

**Published:** 2025-11-17

**Authors:** Mirriam Mkhize, Claire van der Westhuizen, Katherine Sorsdahl

**Affiliations:** Alan J. Flisher Centre for Public Mental Health, Department of Psychiatry and Mental Health, University of Cape Town, South Africa; Makerere University CHS: Makerere University College of Health Sciences, UGANDA

## Abstract

Depression among adolescents is a global concern, including in South Africa, emphasizing the need for reliable screening tools. While the the Patient Health Questionnaire Adolescent (PHQ-A) and Center for Epidemiologic Studies - Depression Scale for Children (CES-DC) are commonly used mental health screening tools among adolescents, their psychometric properties in South Africa are not well-studied. This study aims to fill this gap by examining the psychometric properties of the PHQ-A and CES-DC among adolescents aged 10–14-year-olds in South Africa. First, a sample of 42 adolescents aged 10–14 years were recruited to participate in cognitive testing focus groups to determine their comprehension of the depression screening tool items. Secondly, a sample of 500 adolescents from 10 schools completed a tablet-based survey including socio-demographic information and the adapted depression screening tools. A subset of 123 adolescents underwent clinical diagnostic interview with the MINI-KID depression modules only. After two weeks, a subset of 145 adolescents had the initial tablet-based survey re-administered for test-retest reliability. Criterion validity was examined using ROC analysis, with the MINI-KID as the reference standard. Of the 500 adolescents surveyed, the majority were female (59.2%), with an average age of 12 years (SD = 1.42). The PHQ-A demonstrated good internal consistency (alpha = 0.82) and moderate test-retest reliability (ICC = 0.56). The CES-DC showed good internal consistency (alpha = 0.86) and moderate test-retest reliability (ICC = 0.68). The PHQ-A displayed good sensitivity (85.7%) and specificity (61.8%). Youden’s index indicated 7.5 as the ideal cutoff score for the PHQ-A. The CES-DC has a sensitivity of 95.2% and specificity of 56.9% and Youden’s index of 21. Factor structure for the PHQ-A included one factor loading for 9 items and 3 factor loading for 8 items; while for the CES-DC factor structure consisted of o three factor loadings. The findings show good psychometric properties of the PHQ-A and CES-DC and contributes to the limited research on mental health assessment tools for younger adolescents in Africa. Testing the psychometric properties of the PHQ-A and CES-DC for this population could help improve early detection and intervention for mental health conditions.

## 1. Introduction

The prevalence of mental health conditions among adolescents is high worldwide, particularly in Africa. Between 10% to 20% of adolescents experience a mental health condition [1,2], contributing 5% of disability-adjusted life years (DALYs) and represent a quarter of all years lived with disability (YLDs) among adolescents aged 10–19 [3]. Despite the limited prevalence research in Africa, reported prevalence of depression and anxiety ranges from 5% to 50% [4–9], but most studies focus on high-risk or older adolescents aged 15 and higher [6,7,10], with studies focusing on younger adolescents underrepresented Data from phase 1 of this study, a recent cross-sectional study of 621 adolescents aged 10–14 years in school settings in South Africa found that 33.5% reported experiencing symptoms of depression [11]. While these studies offer valuable insight into symptoms of depression and anxiety among this population, a major limitation is the use of tools that have not been assessed for reliability and validity within the context.

Although the gold standard for identifying adolescents with a mental health condition are structured clinical interviews administered by a trained mental health specialist, resource constraints and a shortage of mental health professionals pose a challenge in low and middle income countries (LMICs) [2,12,13]. Therefore, there is a need for validated, reliable, and culturally-appropriate assessment tools to screen mental health symptoms for children and adolescents [14]. According to a systematic review, the psychometric properties of only 11% of the existing screening tools in LMICs have been tested for use among children and adolescents [15]. Most of the available studies validating screening tools for depression were conducted in high-income countries (HICs) [16,17], with the most utilized screening tool being the Patient Health Questionnaire Adolescent (PHQ-A) [18] and the Center for Epidemiological Studies Depression Scale for children (CES-DC) [19,20]. The PHQ-A is a self-report tool adapted from the PHQ-9 and used to identify depression in adolescents aged 10–18 years [18,21]. However, only three studies all targeting older adolescents have been conducted in Africa [10,22–26]. A recent study examined the isiXhosa version of the PHQ-9 tool among 302 adolescents aged 10–19 in schools [24]. The study revealed an acceptable criterion validity with 91% sensitivity and 76% specificity in identifying adolescents with depression, based on a cut-off score of 10 or higher indicating depression [24]. Apart from this study, there are no others that have examined the psychometric properties of the PHQ-A among adolescents aged 10–14 years.

The Center for Epidemiologic Studies-Depression Scale (CES-D) has been modified for children and adolescents as the CES-DC [27]. It is a 20-item self-report tool designed for adolescents aged 6–17 [19,20]. While the CES-DC has been primarily tested for its psychometric properties in HICs [20,28], there is one study that includes data from Africa [29], with an internal consistency of α = .89 and sensitivity of 80%. However, there are limited data on the CES-DC’s psychometric properties in LMICs particularly in Africa. To test the criterion validity, a study in Rwanda found that the CES-DC is a good tool for depression screening among adolescents aged 10–17, with good internal consistency (α = .86) and test-retest reliability (r = .85) [30]. In South Africa, limited studies have used the CES-DC measure and reported on its criterion validity and reliability [31,32]. This study conducted in the Free State Province sampled 435 South African adolescents aged 13–17 using the CES-DC tool and found good internal consistency (α = .89) [32]. Although some studies have utilized the CES-DC tool to assess depression in older adolescents, there is limited research on its criterion validity and psychometric properties in the local context for South African adolescents aged 10–14 years.

In summary, despite the growing use of the PHQ-A and the CES-DC, very few studies in Africa have examined the psychometric properties of these measures [33,34] in the younger adolescent population group [35]. Existing self-report screening tools often target older adolescents, while for those under 14 years, researchers rely on parent and teacher reports [36,37]. To fill this gap, this study aimed to evaluate the psychometric properties of the self-report PHQ-A and CES-DC for application among South African adolescents aged 10–14 years.

## 2. Materials and methods

To examine the psychometric properties of the PHQ-A and the CES-DC, this study was conducted in two phases. During the first phase, we conducted cognitive interviewing focus groups to assess the participants comprehension of the measures. In the second phase, when the measures had been adapted and finalized, a larger survey was conducted to investigate the psychometric properties of the PHQ-A and CES-DC.

### 2.1. Setting

We collaborated with three non-profit organizations currently working in the Western Cape province, South Africa. For phase one, one school was approached; for phase 2, 10 schools were approached and assisted with recruitment of participants [11]. These schools are based in under resourced areas of the Cape Town metropole and surrounding areas.

### 2.2. Phase 1 recruitment of participants for cognitive testing

We received approval from the University of Cape Town Human Research Ethics Committee (HREC: 565/2020) and the Western Cape Education Department (20211018–6776). In collaboration with one of our partner schools, we recruited a sample of 42 learners for the cognitive testing phase. Participants were selected based on the following criteria: a) aged 10–14; b) enrolled in grades 4–7; and c) provided parental consent and adolescent assent.

### 2.3. Study procedure

To conduct the cognitive testing phase using a focus group discussion (FGD) format, learners in grades 4–7 were identified and approached by school staff. Once all learners were identified the researcher explained the study and the cognitive testing process to all the learners. The consent and assent forms were sent to all their guardians. Learners that returned their signed guardian consent and assent forms were recruited into the study at a convenient time at the school. The PHQ-A and CES-DC were administered to the 42 learners with four focus group discussions (FGD) held with learners. Each focus group session was led by two facilitators, including a social worker and a psychologist. Learners were grouped by grade levels [4–7], with 10–12 learners in each group. During the FGD, the participants’ comprehension of and response to questions were elicited guided by the structured discussion guide for cognitive testing [38]. Learners shared their perspective on the PHQ-A and CES-DC using a structured interview guide. After the session, learners received a gift and a snack box as a thank you for their participation.

### 2.4. Measures

*Depression.* The Patient Health Questionnaire for Adolescents (PHQ-A) is an adolescent version of the Patient Health Questionnaire – 9 item and is used as a screening measure to detect depression among adolescents aged between 10 and 18 years [18,21]. It is self-administered and consists of the nine criteria used in the DSM-IV diagnosis of depressive disorders [21]. The total score can range from 0 to 27, with a suggested cut-off score of 10 and higher indicating greater severity of depression.

The Center for Epidemiologic Studies – Depression Scale for Children (CES-DC) for children and adolescents is a 20 item self-report questionnaire for children and adolescents between the ages of 6 and 17 [19,20]. The total score can range from 0–60, with a suggested CES-DC cutoff score is 15 as being suggestive of depressive symptoms in children and adolescents [28]. That is, scores over 15 can be indicative of significant levels of depressive symptoms [28].

### 2.5. Results of cognitive testing

The researcher analyzed and organized the responses from the cognitive testing FDGs. Minor changes were made to the PHQ-A and CES-DC after the FGD cognitive testing based on the participant’s feedback to improve understanding, language, and wording. Three expert researchers were involved in reviewing and providing feedback on the adjustments before the next phase. The adapted measures were then included into the study survey conducted across the schools in collaboration with our partner schools [11]. Table 1 below illustrate the adjustments made to the PHQ-A and CES-DC (see S1 and S2 Tables).

**Table 1:** PHQ-A original and revised items for use.

Items	Original PHQ-A items	Revised PHQ-A items
**PHQ-A Item 1**	Feeling down, depressed, irritable, or hopeless?	Feeling down(or sad), depressed, irritated, or having no hope?
**PHQ-A Item 2**	Little interest or pleasure in doing things?	Little interest or enjoyment in doing things?
**PHQ-A Item 4**	Poor appetite, weight loss, or overeating?	Not wanting to eat, losing weight or eating too much
**PHQ-A Item 8**	Moving or speaking so slowly that other people could have noticed? Or the opposite—being so fidgety or restless that you were moving around a lot more than usual?	Moving or speaking so slowly that other people could have noticed? Or the opposite—finding it so hard to keep your hands and body still, that you were moving around a lot more than usual?
**CES-DS Item 20**	It was hard to get started doing things.	It was hard to start doing things (e.g., such as schoolwork, chores at home, playing with friends/siblings).

### 2.5. Phase 2: Recruitment of participants for the study survey

Recruitment for phase 2 followed the same process for phase 1. Ten schools were approached and 621 participants were recruited between the ages of 10 and 14 years and data collected between February 2022 and July 2022 [11]. This study was part of a larger study investigating the prevalence and factors associated with depression and anxiety among young school-going adolescents in the Western Cape Province of South Africa [11]. Each participant was provided with a tablet to complete the survey using the user-friendly REDCap platform. The participants choose their preferred language to complete the survey, which was available in English [11]. The survey itself took approximately 45–60 minutes to administer, and an energizer activity was facilitated by the research team halfway through the allotted time [11]. The survey obtained a high response rate from participants (n = 504) who completed it in English out of 621 participants. For this paper, only 500 of the 504 participants are included in this analysis. Four participants were removed owing to missing data.

A team of trained clinical research assistants (including social worker, psychologist, and registered counsellor) administered the depression modules of the Mini Neuropsychiatric Interview version 7.0 (MINI) for Children and Adolescents (MINI-KID) in English to 123 leaners (approximately 5 per class). These 123 participants were randomly selected based on their consent, not their PHQ-A and CES-DC scores. The researchers offered the participants the option to participate in the clinical interview before, during, or after completing the survey.

To assess the test-retest reliability of the PHQ-A and CES-DC, a sample of 145 learners who had provided consent were selected two weeks after the initial administration of the survey. The test–retest interval of two weeks was selected, consistent with standard psychometric practice to provide substantial time to minimize recall bias and limit the likelihood of clinically significant changes in depressive symptoms among adolescents [25,39]. Participants were asked to complete sociodemographic questions, the PHQ-A and CES-DC measures only. All visits included refreshments, and all participants received a snack box for their participation.

### 2.6. Phase 2 measures

Alongside the PHQ-A and CES-DC mentioned previously, the survey included sociodemographic questions relating to participant sex, age, home language and level of education.

*Clinical Diagnosis.* The Mini Neuropsychiatric Interview version 7.0 (MINI) for Children and Adolescents (MINI-KID) [40], was designed as a brief structured diagnostic interview for the major psychiatric disorders in Diagnostic Statistical Manual of mental disorders (DSM-III-R, DSM-IV, and DSM-5) and International Classifications of Disease (ICD-10) psychiatric disorders to assess the 30 most common and clinically relevant disorders [40,41]. The MINI-KID depression module was utilized in this study. Although developed for use in HICs, the MINI-KID has been widely utilized in LMICs as a gold standard for clinical diagnosis of anxiety and depression among children and adolescents [34,42–44] including in Africa [5,30] and South Africa [45–47].

## 3. Data analysis

The survey received a high response rate from participants who completed it in English. As a result, this analysis concentrates solely on the English responses. The Statistical Package for Social Science (SPSS) (29.0) was used to analyse the data. Frequency distributions and descriptive statistics were calculated for the sociodemographic variables of the sample. Receiver operating characteristic (ROC) analysis was performed to evaluate the criterion validity of the CES-DC and the PHQ-A, using the MINI-KID as the gold standard. The coordinates of this curve were tabulated in SPSS, indicating the sensitivities and specificities at various cut-off points. Thereafter the Youden’s index was calculated and included on the table with the sensitives and specificities.

To evaluate the construct validity of the PHQ-A, the principal component analysis (PCA) with varimax rotation was conducted. The researcher first ensured that the underlying assumptions of PCA were met which included: Evaluating the data for normality, linearity, outliers, and factorability [48]. Normality was assessed by visually inspecting histograms and analyzing skewness and kurtosis. Linearity was assessed through scatterplot examinations of variable pairs to ensure a linear relationship. To assess normality, we conducted the Shapiro-Wilk test on each variable and used Q-Q plots to visualize distributions, confirming approximate normality. No outliers were detected for continuous variables, and box plots were produced. Second, we evaluated the sampling adequacy and factorability of the dataset using the Kaiser-Meyer-Olkin (KMO) measure. For the PHQ-A, the KMO value was.896, exceeding the recommended threshold of.6 [49]. Bartlett’s Test of Sphericity yielded a significant result (χ2 (36) = 1295.47, p < .001), indicating the data’s suitability for factor analysis and the sample size adequacy (n = 504) for PCA. Furthermore, we examined multicollinearity by calculating the correlation matrix and ensuring significant inter-correlations among variables without redundancy. After meeting these assumptions, we proceeded with principal component analysis (PCA) and opted for varimax rotation with Kaiser Normalization to extract factors based on eigenvalues >1 [48,50]. The factors explained over 63% of the variances, and we used a scree plot to retain factors before eigenvalues leveled off. Items with communalities between 0.0–0.3 were considered, and only those with factor loadings less than 0.30 were suppressed. Only items with factor loadings of an absolute value of 0.30 or greater were considered, and those instances where there were cross-loadings with a difference between factor loadings of less than 0.2, were considered [49].

The same steps were taken to evaluate the CES-DC PCA assumptions [48], and all criteria were met, including an adequate sample size (n = 504), absence of outliers (confirmed through a box plot), no missing data, and no multicollinearity. Sampling adequacy and factorability were assessed using the Kaiser-Meyer-Olkin (KMO) measure for the CES-DC, resulting in a value of.929. Bartlett’s Test of Sphericity was significant (χ2 (190) = 3510.70, p < .001), indicating the data’s suitability for factor analysis. Factor extraction parameters included eigenvalues >1, factors explaining more than 57% of variances and using a scree plot analysis to retain factors before eigenvalues leveled off. Items with communalities ranging from 0.0–0.3 were considered. Only items with factor loadings above 0.3 were considered and cross-loadings, where the difference between factor loadings was less than 0.2 were also considered [49].

Cronbach’s coefficients were used to evaluate the internal consistency of the PHQ-A, and CES-DC tools. To assess test-retest reliability, we calculated the intraclass correlation coefficient (ICC) to estimate the stability of PHQ-A and CES-DC scores in the first and second administrations using one-way random effects ICC.

## 4. Results

### 4.1. Sociodemographic characteristics

The socio-demographic characteristics of the sample are shown in Table 2. Out of all the participants, 504 (81.2%) decided to complete the survey in English, while 105 (16.9%) completed it in isiXhosa and only 12 (1.9%) in Afrikaans. Of the total of 500 adolescents surveyed, the majority were female (59.2%) with an average age of 12 years (SD = 1.42). More than two-thirds of the participants reported having a dual home language, with 395 (71.8%) speaking English, 214 (42.8%) speaking isiXhosa, 103 (20.6%) speaking Afrikaans, and 58 (11.6%) speaking another language. The participant’s family structure was complex, with participants reporting multiple living arrangements which indicated that more than half 364 (58.6%) live with a single parent, while more than a quarter 162 (26.1%) live with both parents and 95 (15.3%) do not live with their parents.

**Table 2:** Socio-demographic characteristics of the sample.

Variables	n	%
**Age (m, sd)**	12.09, 1.45	
10 years	111	22.2
11 years	94	18.8
12 years	105	21.0
13 years	95	19.0
14 years	95	19.0
**Gender**		
Female/Girl	296	59.2
Male/Boy	204	40.8
**Home language**		
English	359	71.8
IsiXhosa	214	42.8
Afrikaans	103	20.6
Other	58	11.6
**Survey language preference**		
English	504	81.2
IsiXhosa	104	16.9
Afrikaans	12	1.9
**Grade**		
Grade 4	74	14.8
Grade 5	88	17.6
Grade 6	93	18.6
Grade 7	65	13.0
Grade 8	180	36.0
**Family structure**		
Living with both parents	127	25.4
Living with single parent	300	60.0
Living with siblings	101	20.2
Living with extended family	412	82.4

### 4.2. Internal consistency and test retest reliability of the PHQ-A and CES-DC

Only the English responses have been included in the analysis for the purposes of this paper. The results show good internal consistency for both measures, with the Cronbach alpha result of the PHQ-A being 0.83, 95% CI [.81,.85], and for the CES-DC 0.88, 95% CI [.87,.90]. as listed in Table 3. The test-retest reliability results for the PHQ-A indicate moderate reliability (ICC = .57, 95% CI.40−.69) [51]. Similarly, the CES-DC findings demonstrate moderate to good reliability (ICC = .65, 95% CI.51 −.74) in line with the PHQ-A results.

**Table 3:** Internal consistency and test retest reliability.

Questionnaire	Internal consistency (n = 500)	95% CI	Test-Retest Reliability (n = 145)
PHQ-A English	0.83	[.81,.85]	0.57 (0.40 - 0.69)
CES-DC English	0.88	[.87,.90]	0.65 (0.51 - 0.74)

#### 4.2.1. ROC curve analysis for PHQ-A.

**Fig 1:**
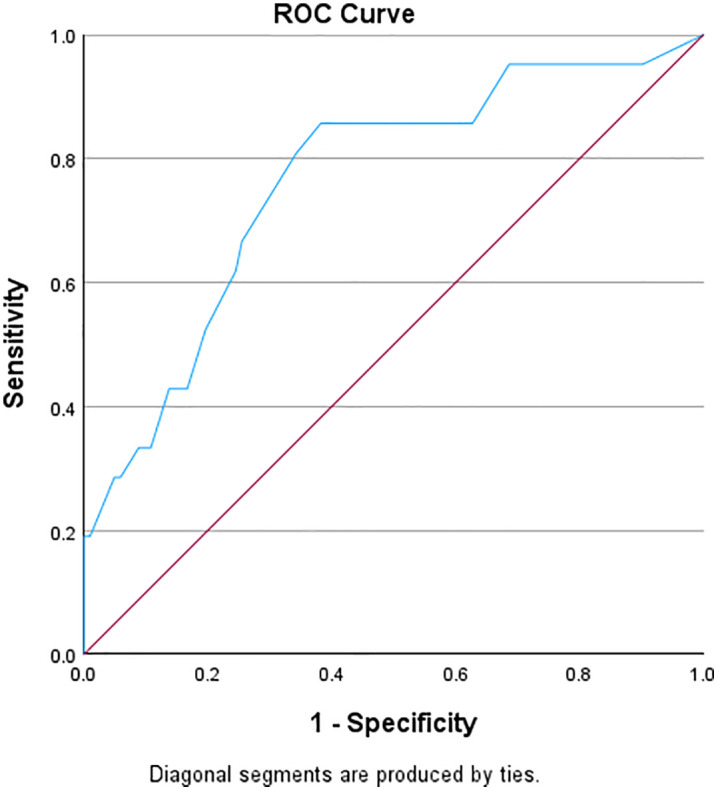
ROC curve for PHQ-A.

The ROC curve analysis evaluated the sensitivity and specificity of PHQ-A against the MINI-KID depression module English only. Table 4 displays the properties of the performance of the PHQ-A ROC analysis. Results correspond to the ROC curve shown in Fig 1.

**Table 4:** Properties of the performance of the PHQ-A ROC curve analysis.

	AUC (95% CI)	Cut-off	Sensitivity	Specificity
PHQ-A	0.764 (0.64-0.87)	7.5	0.85	0.47

The Table 4 shows that a Youden’s index of 0.475 is highest at a cut-off score of 7.50 (rounded off to 8), suggesting that this score optimally balances sensitivity and specificity for the PHQ-A. Therefore, the recommended cut-off score, based on Youden’s index is 7.5 (rounded off to 8) for this context based on the results on ROC curve analysis as depicted in Fig 1, with a sensitivity of 85.7% and specificity of 61.8%. This suggests that the PHQ-A is fairly sensitive meaning it correctly identifies 86% of true positive case while it has a moderate specificity, correctly identifying 62% of true negative cases. Furthermore, the area under the curve (AUC) value of 0.764 suggests that the ROC curve, based on the PHQ-A, shows good discriminatory ability between the negative and positive cases.

#### 4.2.2. ROC curve analysis for CES-DC.

**Fig 2:**
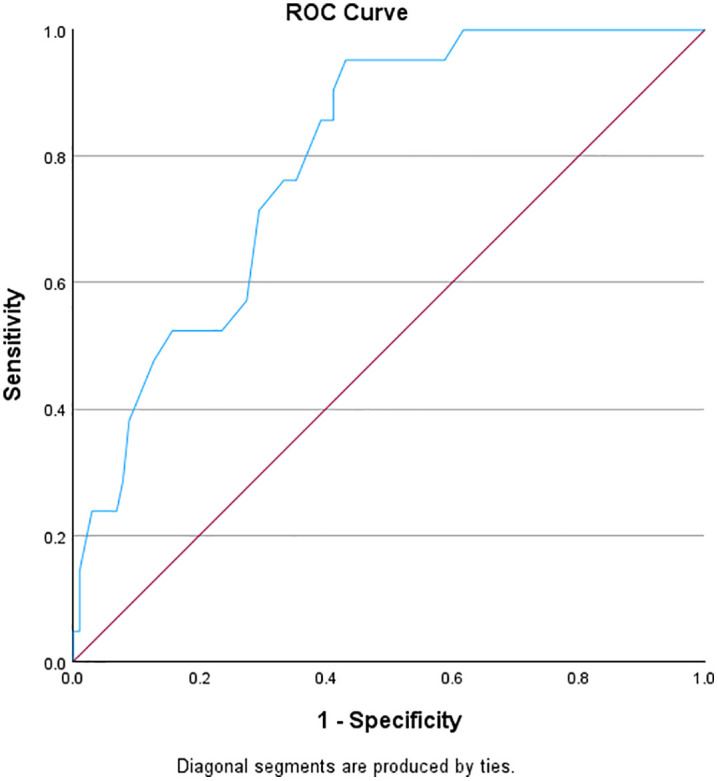
ROC curve for CES-DC.

The ROC curve analysis evaluated the sensitivity and specificity of CES-DC against the MINI-KID depression module. Table 5 displays the properties of the performance of the *CES-DC ROC* curve analysis. Results correspond to the ROC curve shown in Fig 2.

**Table 5:** Properties of the performance of the CES-DC ROC curve analysis.

	AUC (95% CI)	Cut-off	Sensitivity	Specificity
CES-DC	0.797 (0.70-0.88)	21.50	0.95	0.52

Table 5 shows that a Youden’s index of 0.521 is highest at a cut-off score of 21.50 (rounded off to 22), suggesting that this score optimally balances sensitivity and specificity for the CES-DC. Therefore, the recommended cut-off score, based on Youden’s index is 21.50 for this context based on the results of the ROC curve analysis as shown in Fig 2, with a sensitivity of 95.2% and specificity of 56.9%. Furthermore, the area under the curve (AUC) value of 0.797 suggests that the ROC curve, based on the CES-DC, shows good discriminatory ability to differentiate between the positive and negative cases.

### 4.4. Factor analysis: Principal component analysis for the PHQ-A

The principal component analysis (PCA) with varimax rotation was conducted to assess the underlying structure of the PHQ-A examining depressive symptoms among the 500 adolescents.

**Fig 3:**
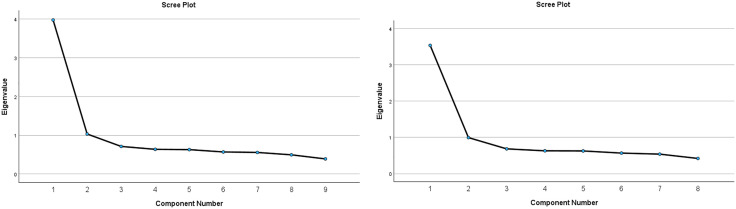
Scree plot factors for the PHQ-A 9 items. Scree plot factors for the PHQ-A 8 items.

For this analysis, we conducted two analyses based on findings from previous research: (i) a PCA analysis including all the adapted PHQ-A 9 items and (ii) a second analysis with the adapted PHQ-A excluding the ninth question, which relates to suicide [52]. The findings from Table 6 and Fig 3 showed the data met the assumptions of sample adequacy (KMO = .89), the absence of multicollinearity, and significance of Bartlett’s test of sphericity. Two factors were extracted from the analysis – ‘depression’ and ‘somatic’. The first component showed high loadings on items relating to low mood, anhedonia, and negative thinking, while the second component showed higher loadings on items reflecting somatic symptoms such as sleep, appetite, and fatigue. The results from Table 7 and Fig 3 PHQ-A 8 items, data met the assumptions of sample adequacy (KMO = .87), the absence of multicollinearity, and significance of Bartlett’s test of sphericity. Only one principal component factor was extracted from the analysis with the removal of item 9. The scree plot confirms the extraction of the one component. A unidimensional principal component factor was indicated with all the principal component factors loaded into one factor.

**Table 6:** Factors derived from the adapted PHQ-A 9 items English only.

Items	Questions	Factor 1	Factor 2
1	Feeling down(or sad), depressed, irritated, or having (no hope)	.68	
2	Little interest or enjoyment in doing things?		.86
3	Trouble falling asleep, staying asleep, or sleeping too much?		.54
4	Not wanting to eat, losing weight or eating too much		.56
5	Feeling tired, or having little energy?		.53
6	Feeling bad about yourself—or feeling that you are a failure, or that you have let yourself or your family down?	.77	
7	Trouble concentrating on things like schoolwork, reading, or watching TV?	.66	
8	Moving or speaking so slowly that other people could have noticed? Or the opposite—finding it so hard to keep your hands and body still, that you were moving around a lot more than usual?	.66	
9	Thoughts that you would be better off dead, or of hurting yourself in some way?	.74	

**Table 7:** Factors derived from the adapted PHQ-A 8 items English only.

Items	Questions	Factor 1
1	Feeling down(or sad), depressed, irritated, or having (no hope)	.74
2	Little interest or enjoyment in doing things?	.40
3	Trouble falling asleep, staying asleep, or sleeping too much?	.69
4	Not wanting to eat, losing weight or eating too much	.69
5	Feeling tired, or having little energy?	.71
6	Feeling bad about yourself—or feeling that you are a failure, or that you have let yourself or your family down?	.71
7	Trouble concentrating on things like schoolwork, reading, or watching TV?	.64
8	Moving or speaking so slowly that other people could have noticed? Or the opposite—finding it so hard to keep your hands and body still, that you were moving around a lot more than usual?	.65

### 4.6. Factor analysis: Principal component analysis for the CES-DC

The principal component analysis (PCA) with varimax rotation was conducted to assess the underlying structure of the adapted 20 items of the CES-DC examining depressive symptoms among the 500 adolescents.

**Fig 4:**
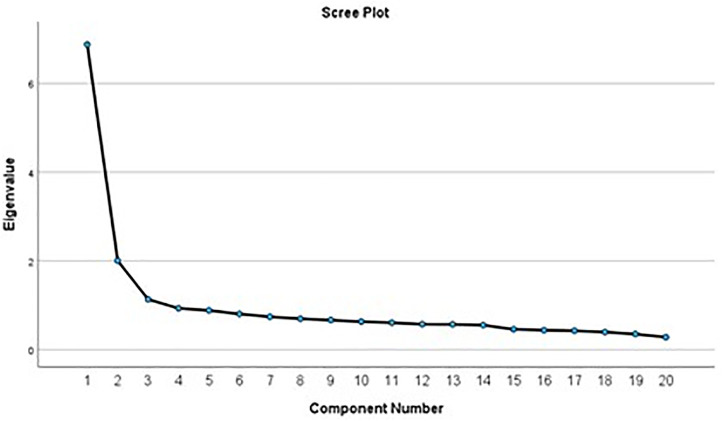
Scree plot factors for the CES-DC.

Table 8 and Fig 4 of the analysis showed the data met the assumptions of sample adequacy (OKM = .92), the absence of multicollinearity, and significance of Bartlett’s test of sphericity. Factor extraction established three components with eigenvalues greater than one accounted for 28.0% of the variation in depressive symptoms among adolescents. Three principal component factors were extracted from which relate to somatic symptoms, depressed mood and positive affect.

**Table 8:** Factors derived from the adapted CES-DC English only.

Items	Questions	Factor 1	Factor 2	Factor 3
1	I was bothered by things that usually don’t bother me	.53		
2	I did not feel like eating, I wasn’t very hungry	.51		
3	I wasn’t able to feel happy, even when my family or friends tried to help me feel better	.48		
4	I felt like I was just as good as other kids.			.60
5	I felt like I couldn’t pay attention to what I was doing.	.67		
6	I felt down and unhappy	.62		
7	I felt like I was too tired to do things.	.65		
8	I felt like something good was going to happen			.75
9	I felt like things I did before didn’t work out right.	.52		
10	I felt scared.		.55	
11	I didn’t sleep as well as I usually sleep.	.66		
12	I was happy.			.74
13	I was more quiet than usual.	.55		
14	I felt lonely, like I didn’t have any friends.		.75	
15	I felt like kids I know were not friendly or that they didn’t want to be with me		.75	
16	I had a good time			.70
17	I felt like crying.		.70	
18	I felt sad.		.69	
19	I felt people didn’t like me.		.67	
20	It was hard to start doing things (e.g., such as schoolwork, chores at home, playing with friends/siblings).	.56		

## 5. Discussion

This study is one of the first to investigate the psychometric properties of the PHQ-A and CES-DC, which have been culturally adapted for adolescents aged 10–14 in school settings in the Western Cape Province of South Africa. Three key findings emerge from this research.

First, the study findings indicate that both the PHQ-A and CES-DC demonstrate good internal consistency and moderate test-retest reliability, suggesting that the items are highly correlated and measure the intended construct reliably among young adolescents in South Africa. Consistent with previous studies conducted in Africa, the South African adapted PHQ-A exhibited acceptable internal consistency [24,26,30,53,54] with a Cronbach’s alpha = 0.83. Notably, compared to a recent study conducted by Lovero and colleagues in 2022 involving 485 adolescents aged 12–19 years in Mozambique, our research identified slightly higher internal reliability for the English PHQ-A (Cronbach’s alpha = 0.83), in contrast to the Mozambican Portuguese language (Cronbach’s alpha = 0.80) [26].

While limited research has explored the internal consistency of the CES-DC in Africa [30,55]. By comparison, the study conducted by Betancourt and colleagues’ in Rwanda using Kinyarwanda language yielded results similar to ours [30]. Their study revealed an internal consistency coefficient of 0.86 for the CES-DC scale [30]. In contrast, although our study demonstrated higher internal consistency (Cronbach’s alpha = 0.88). The findings emphasize good internal consistency across the studies conducted among young African adolescents, with our study reporting slightly higher internal consistency.

Test-retest reliability is another aspect of reliability, as it assesses the consistency of scores over time, reflecting the stability of measurements. The moderate test-retest reliability coefficients obtained for the PHQ-A (ICC = 0.57) and the CES-DC (ICC = 0.65) suggest that these screening tools yield relatively consistent results when administered at different time points. Previous studies have also shown stability in scores for the PHQ-A and CES-DC over time [21,27,56]. By contrast, our study findings differ from those of Lovero’s study in Mozambique where they reported a good test-retest reliability coefficient of 0.70 for the PHQ-A [26], our research identified poor to moderate test-retest reliability for the PHQ-A (ICC = 0.57). Similarly, for the CES-DC, our study revealed moderate test-retest reliability (ICC = 0.65) in comparison to the good Pearson coefficient of 0.85 for test-retest reliability among Rwandan adolescents [30].

These findings underscore the impact of cultural influences as a potential source of variation in our findings. This is reflected in the high internal consistency of the depression screening tools and differences in test-retest reliability, emphasizing the need to consider both cultural and linguistic factors in research and mental health screening and assessment [26,57,58].

Although our study reported on the English PHQ-A and CES-DC responses, it is crucial to address the translation process of depression screening tools from English to African languages. After conducting cognitive interviews, several items were reworded to better reflect the psychological construct and improve linguistic and cultural clarity. This careful adaptation is a crucial step in cross-cultural research, as making a screening tool more comprehensible increases its validity when used in diverse contexts. To address this, it is important to consider a rigorous translation methodology and conduct cognitive testing interviews to check consistency of interpretation of the translation with the original language. The translation process may influence internal consistency, potentially leading to variations in tool interpretation and responses. This aligns with validation studies conducted in Kenya and Nigeria, emphasizing the significance of multiple language considerations [59,60].

The second finding of our study is the ROC curve analysis, which assessed the sensitivity and specificity of the PHQ-A and CES-DC against the MINI-KID depression module among adolescents in South Africa. Regarding criterion validity, our findings for the PHQ-A revealed a recommended cut-off score of 7.50 (rounded off to 8), based on the highest Youden’s index of 0.475, which balances sensitivity and specificity for this context. The sensitivity of 85.7% and specificity of 61.8% indicate that the PHQ-A is fairly sensitive in correctly identifying true positive cases, while also demonstrating moderate specificity in correctly identifying true negative cases. The Area Under the Curve (AUC) value of 0.764 suggests that the ROC curve for the PHQ-A shows good discriminatory ability in distinguishing between negative and positive cases of depressive symptoms among adolescents in South Africa.

Our findings are consistent with prior research. For example, Lovero and colleagues’ study also reported good sensitivity and specificity values (>0.70), and the Youden’s index identified an optimal cutoff score of 8 for the PHQ-A [26]. In contrast, our results differ from other studies conducted in South Africa [24], Kenya [54] and Nigeria [60], where good to high sensitivity and specificity values (>0.70–0.90) were observed. All three studies identified an optimal cutoff score of >10 or higher [24,54] compared to our findings.

The ROC curve analysis of the CES-DC identified a recommended cut-off score of 21.50 (rounded off to 22), based on the highest Youden’s index of 0.521, which optimally balances sensitivity and specificity for this screening tool. The sensitivity of 95.2% and specificity of 56.9% indicate that the CES-DC is highly sensitive in correctly identifying true positive cases, while showing moderate specificity in correctly identifying true negative cases. The AUC value of 0.797 suggests that the ROC curve for the CES-DC demonstrates good discriminatory ability in distinguishing between positive and negative cases of depressive symptoms among adolescents in South Africa. In contrast to our findings, a previous study assessed the CES-DC’s psychometric properties in Rwandan adolescents aged 10–19 using the MINI-KID clinical interview reported indicated that a CES-DC score of ≥ 30 yielded a sensitivity of 0.82 and a specificity of 0.72 in detecting depression, with an AUC of 0.83 [30]. The optimal score was found to be > 30, which is higher than the > 21 reported in our study, along with different sensitivity and specificity values compared to our findings.

The sensitivity, specificity, and discriminatory ability of these screening tools ensure their applicability for early detection and intervention of mental health conditions in this population. Early detection enables timely management of depressive symptoms among adolescents in clinical and school settings. The high sensitivity rate observed in our study plays a crucial role in achieving this objective by accurately identifying true positives and reducing the risk of false negatives [58]. It is important to note the potential for over identification of false positives due to moderate specificity in our results. Nonetheless, the specificity values were moderate (i.e., 56–62%) with context-dependent considerations. The clinical context may lead to lower specificity generating more false positives and adding burden to systems with limited resources; this might be navigated by means of a two-stage pathway (brief screening followed by a clinician interview or a second validated measure administered by trained clinicians or non-specialists) and context-adjusted cut-offs [58,61]. In the context of screening in schools and communities, where the objective is to avoid missing at-risk adolescents, lower specificity is a reasonable trade-off as long as positive screens are followed by organized secondary assessment and clear referral options [58,61]. However, in our context where early identification is paramount, this can be managed through strategies such as additional follow-up assessments to confirm diagnoses or considering the context of use, whether for initial mental health screening or comprehensive diagnostic evaluations [58,61].

The third finding of this study from the PCA analysis provide valuable insights into the factor structure of the PHQ-A and CES-DC screening tools. The PCA analysis of the PHQ-A revealed two distinct factors: depression and somatic symptoms. This finding is consistent with previous research that has identified similar factor structures in adolescent populations [25,62]. The extraction of these two factors suggests that the PHQ-A captures both emotional and physical symptoms of depression among adolescents in South Africa. The removal of the ninth item related to suicide from the analysis resulted in a unidimensional factor, indicating that this item may not align with the underlying structure of depressive symptoms in this population. However, these findings are consistent with previous studies indicating that item 9, assessing self-harm and suicidal thoughts, does not effectively differentiate between individuals likely to be depressed and those who are not, and also exhibits a lower factor loading [21,63]. Contrary to previous studies using all 9 items, suggesting a single factor loading for the PHQ-A, our study revealed two factor loadings for the PHQ-A 9 items [21,64]. Nevertheless, item 9 of the PHQ-A which measures self-reported self-harm or suicidal ideation, may be influenced by cultural norms and stigma, leading adolescents to underreport due to fear, shame or stigma [65]. Meanwhile, it is also observed that by contrast some adolescents tend to misunderstand what constitutes suicidal ideation, leading to a potentiality of false positive and burdening scarce clinical resources [66–68]. Therefore, these findings underscore the need for culturally sensitive administration by trained clinicians or non-specialists, in-depth understanding of each item meaning and structured clinical interviews to validate positive responses.

In contrast, the PCA analysis of the CES-DC identified three components: somatic symptoms, depressed mood, and positive affect, showcasing the multidimensional nature of depressive symptoms captured by the screening tool. This contrasts with prior research that reported a four-factor loading for the CES-DC to include: somatic complaints, depressed affect, interpersonal problems and positive affect [27,55,69]. By contrast, our findings reported three factor loadings which include somatic symptoms, depressed mood, and positive affect indicate that the CES-DC offers a comprehensive assessment of different symptoms of depression among adolescents. These differences underscore the importance of conducting PCA analyses in specific cultural contexts to understand the unique factor structure of screening tools for mental health assessment.

The findings of this study underscore important implications for practice, particularly suggesting that the PHQ-A could serve as a screening tool in schools and possibly in primary care settings. Its brevity and alignment with DSM criteria for depression symptoms make it suitable for use by trained clinicians,(e.g., lay counsellors or social workers). In school or community settings, a trained lay counsellor can employ this tool, provided there are adequate referral pathways for adolescents identified as at-risk. However, these implications should be interpreted in light of several limitations. First, only the English version of the instrument underwent cognitive testing and validation, introducing possible language bias of participants who preferred their home language (e.g., isiXhosa or Afrikaans). Second, the sample was drawn from the Western Cape, the findings may not be generalisable to school-going adolescents in other South African provinces other provinces in South Africa and other LMICs contexts. Third, while interviewers were blinded to PHQ-A scores, potential biases inherent to interviewer-administered diagnostics (e.g., interviewer effects) cannot be fully excluded, which may have introduced bias in the diagnostic interview process.

## 6. Conclusion

Despite the limitations, our findings provide insight on the reliability, validity, and psychometric properties of the PHQ-A and CES-DC screening tools for detecting depressive symptoms among adolescents aged 10–14 years in South Africa. These findings add to the sparse literature on mental health assessment among adolescents, emphasizing the significance of culturally relevant and reliable screening tools for this population. Furthermore, they assist in promoting early detection and intervention for adolescent mental health conditions. Further research is needed to validate these findings in larger and more diverse populations. By improving our understanding of the psychometric properties of these screening tools, we can better support the mental health of adolescents in South Africa and other LMICs contexts. Finally, these tools also hold potential for integration into school health programs and broader mental health systems, where early identification and intervention can be embedded within existing structures to promote scalable and sustainable impact.

## Supporting information

S1 Table Original and modified PHQ-A items. (DOCX)

S2 Table Original and modified CES-DC items. (DOCX)
