# Supplementary material for: Investigating the psychometric properties of the Patient Health Questionnaire for Adolescents (PHQ-A) and Center for Epidemiologic Studies - Depression Scale for Children (CES-DC) among young adolescents in South Africa
Source: PLoS One. 2025 Nov 17;20(11):e0334658. doi: 10.1371/journal.pone.0334658 (PMC12622798; doi:10.1371/journal.pone.0334658)
Supplement: S1 Table — (DOCX) [file pone.0334658.s001.docx]

# S1 Table original and modified PHQ-A items

| **Original Items** | **Adapted items** |
| --- | --- |
| 1. Feeling down, depressed, irritable, or hopeless? | 1. Feeling down(or sad), depressed, irritated, or having no hope ? |
| 1. Little interest or pleasure in doing things? | 2. Little interest or enjoyment in doing things? |
| 1. Trouble falling asleep, staying asleep or sleeping too much? | 3. Trouble falling asleep, staying asleep or sleeping too much? |
| 1. Poor appetite, weight loss, or overeating? | 4. Not wanting to eat, losing weight or eating too much |
| 1. Feeling tired, or having little energy? | 5. Feeling tired, or having little energy? |
| 1. Feeling bad about yourself or feeling that you are a failure or that you have let yourself or your family down? | 6. Feeling bad about yourself or feeling that you are a failure or that you have let yourself or your family down? |
| 1. Trouble concentrating on things like schoolwork, reading or watching TV? | 7. Trouble concentrating on things like schoolwork, reading or watching TV? |
| 1. Moving or speaking so slowly that other people could have noticed? Or the opposite—being so fidgety or restless that you were moving around a lot more than usual? | 8. Moving or speaking so slowly that other people could have noticed?  Or the opposite—finding it so hard to keep your hands and body still, that you were moving around a lot more than usual? |
| 1. Thoughts that you would be better off dead or hurting yourself in some way? | 9. Thoughts that you would be better off dead or hurting yourself in some way? |
