# Supplementary material for: Investigating the psychometric properties of the Patient Health Questionnaire for Adolescents (PHQ-A) and Center for Epidemiologic Studies - Depression Scale for Children (CES-DC) among young adolescents in South Africa
Source: PLoS One. 2025 Nov 17;20(11):e0334658. doi: 10.1371/journal.pone.0334658 (PMC12622798; doi:10.1371/journal.pone.0334658)
Supplement: S2 Table — (DOCX) [file pone.0334658.s002.docx]

# S2 Table original and modified CES-DC items

| **Original items** | **Adapted Items** |
| --- | --- |
| 1. I was bothered by things that usually don’t bother me | I was bothered by things that usually don’t bother me |
| 1. I did not feel like eating, I wasn’t very hungry | I did not feel like eating, I wasn’t very hungry |
| 1. I wasn’t able to feel happy, even when my family or friends tried to help me feel better | I wasn’t able to feel happy, even when my family or friends tried to help me feel better |
| 1. I felt like I was just as good as other kids. | I felt like I was just as good as other kids. |
| 1. I felt like I couldn’t pay attention to what I was doing. | I felt like I couldn’t pay attention to what I was doing. |
| 1. I felt down and unhappy | I felt down and unhappy |
| 1. I felt like I was too tired to do things. | I felt like I was too tired to do things. |
| 1. I felt like something good was going to happen | I felt like something good was going to happen |
| 1. I felt like things I did before didn’t work out right. | I felt like things I did before didn’t work out right. |
| 1. I felt scared. | I felt scared. |
| 1. I didn’t sleep as well as I usually sleep. | I didn’t sleep as well as I usually sleep. |
| 1. I was happy. | I was happy. |
| 1. I was more quiet than usual. | I was more quiet than usual. |
| 1. I felt lonely, like I didn’t have any friends. | I felt lonely, like I didn’t have any friends. |
| 1. I felt like kids I know were not friendly or that they didn’t want to be with me | I felt like kids I know were not friendly or that they didn’t want to be with me |
| 1. I had a good time. | I had a good time. |
| 1. I felt like crying. | I felt like crying. |
| 1. I felt sad. | I felt sad. |
| 1. I felt people didn’t like me. | I felt people didn’t like me. |
| 1. It was hard to start doing things. | It was hard to start doing things (e.g. such as schoolwork, chores at home, playing with friends/siblings). |
